# Adrenal Abscess in a Chronic Hemodialysis Patient: A Case Report

**DOI:** 10.7759/cureus.49497

**Published:** 2023-11-27

**Authors:** Selma Khouchoua, Kaoutar Imrani, Zaynab Iraqi Houssaini, Nabil Moatassim Billah, Ittimade Nassar

**Affiliations:** 1 Radiology Department, Ibn Sina University Hospital, Mohamed V University, Rabat, MAR; 2 Radiology Department, Ibn Sina University Hospital, Mohammed V University, Rabat, MAR

**Keywords:** hemodyalisis, mass, mri, ct, sepsis, abscess, adrenal gland

## Abstract

Adrenal gland abscesses are rare lesions usually reported to be caused by fungal pathogens and typically through hematogenous spread from other primary sources of infection. Imaging has always been known to play a major role in the characterization of focal adrenal lesions. However, given the rare occurrence of abscesses in this location, making the right diagnosis remains challenging. We report the case of a 39-year-old man with chronic renal disease on hemodialysis presenting with signs of sepsis and left upper quadrant pain revealing a left adrenal gland abscess.

## Introduction

Adrenal gland abscesses are very rare lesions occurring in the adrenal gland, with only a few cases reported in the literature. Bacteriemia and hematogenous spread is the most likely cause of adrenal abscesses. Although it remains challenging, imaging with computed tomography and magnetic resonance imaging plays a major role in making the correct diagnosis.

## Case presentation

The present case is for a 39-year-old man with chronic renal disease on hemodialysis presenting with abdominal pain, fever, chronic fatigue, and weight loss. Physical examination found a tachycardic patient with a palpable tender mass in the left flank. Laboratory results found an elevated white blood cell count at 23400/mm3, a high C-reactive protein at 187 mg/L, and positive hemocultures. An abdominal ultrasound was initially performed, showing small kidneys, no hydronephrosis, and a large heterogeneous mass next to the upper pole of the left kidney.

**Figure 1:**
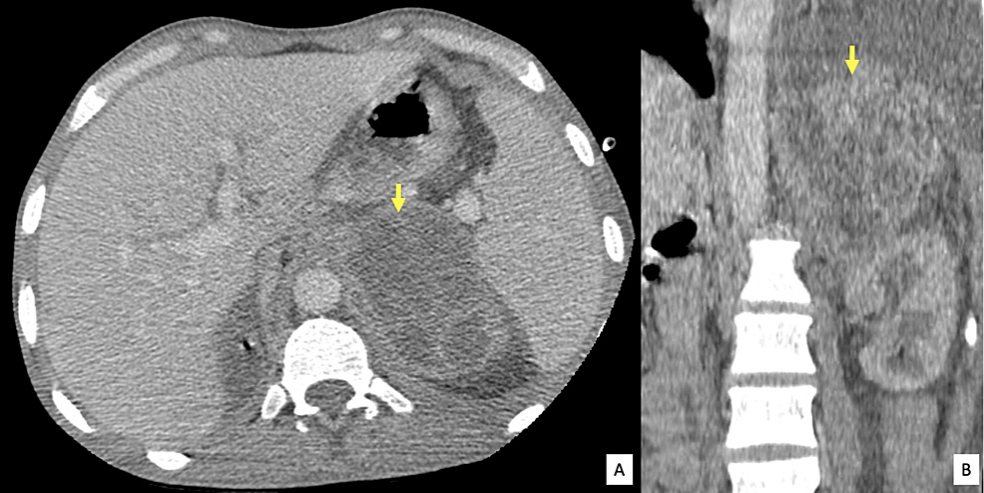
Axial (A) and coronal (B) CT images Left adrenal mass with lobulated margins and heterogenous enhancement with no calcifications and no invasion of the adjacent upper pole of the kidney, medial aspect of the spleen, or diaphragm (arrows).

A thoracic and abdominopelvic computed tomography was performed for further evaluation of the previously described mass and to determine the origin of the sepsis. It showed a large retroperitoneal mass arising from the left adrenal gland. This mass had lobulated margins, heterogenous density with multiple low attenuation cystic non-enhancing areas, with no calcifications (Figure 1).

**Figure 2:**
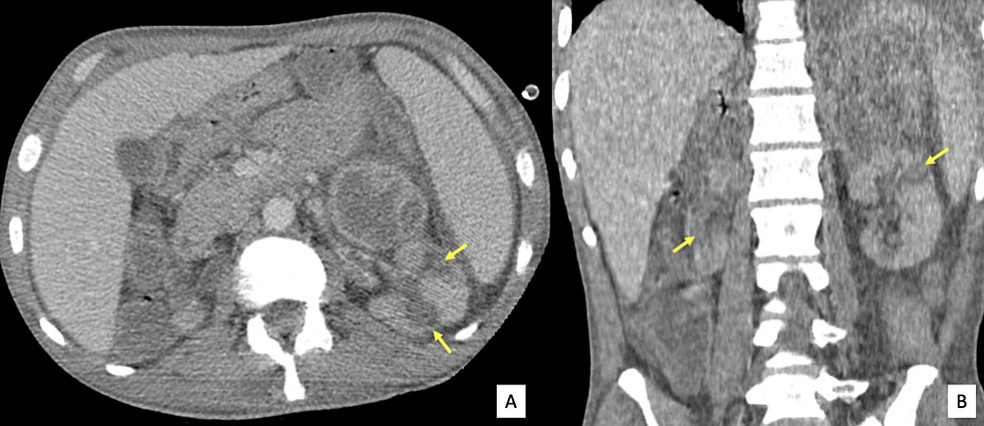
Axial (A) and coronal (B) CT images Wedge-shaped hypodense lesions in both kidneys (arrows) along with perinephric fat stranding.

The mass showed a heterogenous enhancement and surrounding fat stranding. No invasion of the adjacent kidney or surrounding pancreas, medial aspect of the spleen, or diaphragm was depicted. Many wedge-shaped hypodense lesions in both kidneys were found along with perinephric fat stranding (Figure 2).

**Figure 3:**
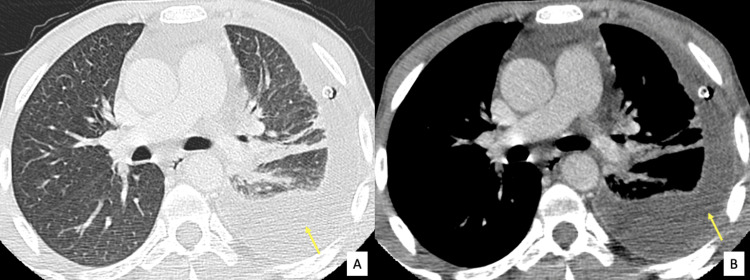
Axial Chest CT in lung parenchyma (A) and mediastinal (B) windows Left pleural effusion with passive atelectasis. No parenchymal consolidation or lung nodules were found otherwise.

Multiple necrotic para-aortic lymphadenopathies were identified. Additionally, there were minimal ascites. No pulmonary nodule or signs of pneumonia was found other than a moderate-sized left pleural effusion (Figure 3).

**Figure 4:**
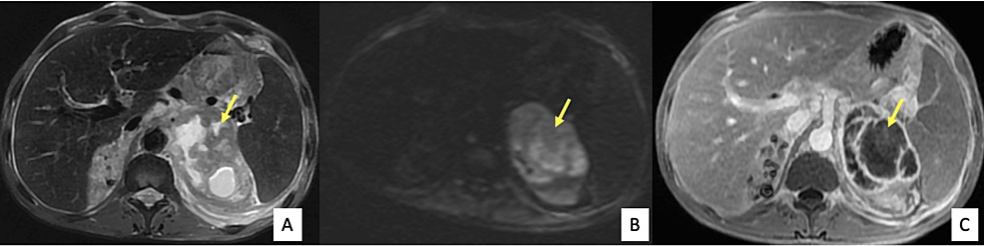
Axial T2 (A), Diffusion (B), and fat-suppressed T1 after gadolinium administration (C) weighted images Multiloculated left adrenal mass mainly cystic in high T2 signal intensity, high signal on diffusion, with a thickened wall and septations in intermediate to low T2 signal intensity with no diffusion restriction and peripheral enhancement after contrast media administration (arrows).

**Figure 5:**
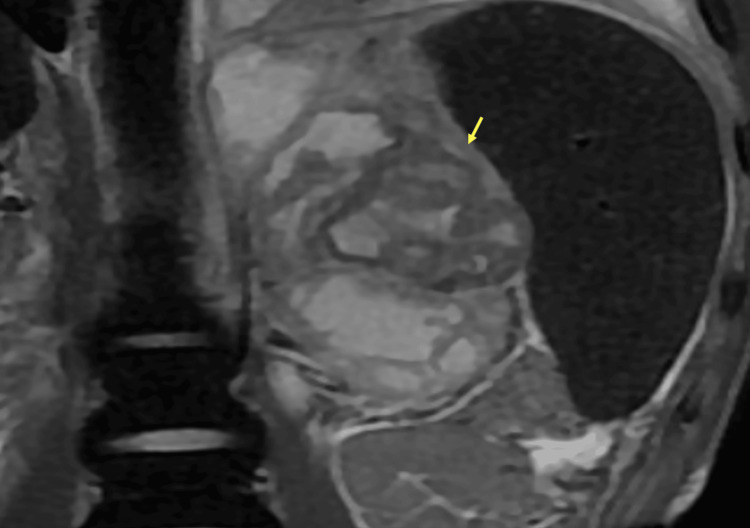
Coronal T2 weighted image Multiloculated left adrenal mass with a thickened wall and septations in intermediate to low T2 signal intensity (arrow).

A further abdominal MRI was performed, which showed a multiloculated adrenal mass with high T2 signal intensity, with no significant diffusion restriction, thickened wall and septations in intermediate to low T2 signal intensity, and peripheral enhancement after contrast media administration (Figures 4, 5).

Given the sepsis, the presence of a multiloculated peripherally enhancing mass and associated signs of nephritis with subsequent perilesional fat stranding, and positive hemocultures, the diagnosis of left adrenal abscess was made. Moreover, pleural fluid analysis showed transudative fluid with no evidence of infection. The patient initially received intravenous probabilistic antibiotics. Percutaneous image-guided drainage was performed and isolated *Mycobacterium tuberculosis*. Subsequently, the patient received oral antibiotics and the treatment regimen consisted of 2 months of isoniazid, rifampin, ethambutol, and pyrazinamide followed by 7 months of rifampin and isoniazid, with overall satisfactory evolution and resolution of symptoms.

## Discussion

Adrenal gland abscesses are very rare lesions occurring in the adrenal gland. Only a few cases are reported in the literature. They usually occur in the setting of bacteriemia [1], hematogenous spread being the most likely cause of adrenal abscesses. The main pathogens to be reported are *Streptococcus pneumoniae *and *Nocardia *[2-4].

Clinical presentation includes signs of sepsis, left or right upper quadrant abdominal pain, and even septic shock.

Computed tomography demonstrates a cystic, usually multi-loculated adrenal mass with peripheral enhancement and possible internal enhancing septations [5]. Surrounding fat stranding is also a typical finding. On MRI, a heterogenous mass with low T1, high T2 signal intensity cystic areas, peripheral enhancement, and possible diffusion restriction are the main findings [6].

Making this diagnosis remains challenging because it is not among the main differentials to consider in the setting of an adrenal lesion. Most of the time adrenal lesions are incidental findings, the most common being the benign incidental adenoma. The wide range of neoplasms involving the adrenal gland varies from primary to metastatic and from benign to malignant. The main diagnoses typically considered are adenomas, pheochromocytomas, and adrenocortical carcinomas. Imaging with computed tomography as the first line imaging modality and magnetic resonance imaging help narrow down the diagnosis. Patterns of enhancement and especially washout evaluation provide valuable information and a roadmap to guide image interpretation. Adenomas typically demonstrate rapid washout, defined as an absolute percentage washout (APW) of more than 60% and a relative percentage washout (RPW) of more than 40% on delayed images. However, adrenocortical carcinomas typically have an RPW of less than 40% [5].

Making the differential between adrenal abscesses and the necrotic changes in an adrenocortical carcinoma can be challenging. The large size and heterogenous appearance are more commonly encountered in malignant lesions. MRI represents a promising imaging modality in distinguishing between a necrotic malignant mass and an adrenal abscess. Typical adrenocortical carcinoma features on MRI include low to iso signal intensity on T1 and intermediate signal intensity on T2 with prevailing heterogeneity due to necrosis and hemorrhage [7,8]. Adrenal abscesses, on the other hand, tend to show markedly T2 hyperintense and diffusion restriction of the cystic component with peripheral enhancement and surrounding fat stranding rather than adjacent organ invasion like in adrenocortical carcinomas. Also, if septations are present, like in our case, they can feature intermediate to low T2 signal intensity and no diffusion restriction, unlike solid components in malignancy. 

Another entity to consider in the current case given the cystic component is cystic adrenal lesions. They are classically divided into pseudocysts, endothelial cysts, epithelial cysts, and parasitic cysts like hydatid cysts. These lesions are known to be asymptomatic, well-demarcated, thin-walled, homogenous cystic lesions with low attenuation values on CT and homogenous, markedly high T2 signal intensity and low T1 signal intensity on MRI [9].

To make a confident diagnosis, it appears crucial to analyze both clinical presentation and imaging findings. In fact, our patient was known to be on hemodialysis, which represents a high risk factor for bacteremia, and he presented with signs of sepsis, along with positive hemocultures. On imaging, the presence of a multiloculated adrenal mass with surrounding fat stranding favored the diagnosis of an adrenal gland abscess. If left untreated it can cause septic shock with high morbidity and mortality rates.

Interventional radiology with percutaneous, image-guided drainage along with infection control using intravenous antibiotic administration adapted to the result of bacterial cultures represents the treatment of choice with very good results and successful outcomes [10].

## Conclusions

Adrenal abscesses remain a rare and challenging diagnosis to make. Both clinicians and radiologists should be aware of this entity. Close attention to clinical presentation and good knowledge of imaging findings are pivotal to making an accurate and timely diagnosis to avoid treatment delays and complications.
